# Methodological and Statistical Comments on T Helper Cytokines Profile and Hepatitis C

**DOI:** 10.5812/hepatmon.10641

**Published:** 2013-06-18

**Authors:** Ali Kabir

**Affiliations:** 1 Nikan Health Researchers Institute, Tehran, IR Iran

**Keywords:** Matched Case Control Studies, Non Parametric Statistics, Healthy Worker Effect, Subgroup Analysis, Power

*Dear Editor,*

I read with much interest paper by Sofian M et al. about serum profile of T helper 1 and 2 cytokines in HCV infected patients (1). They have presented that “unfortunately, some immunological indicators are not normally distributed, and this can minimally affect the results”. Simply, they could use Mann-Whitney U instead of t test and Kruskal-Wallis H instead of ANOVA so they would be able to have correct results specifically when the sample size is small and also the distribution is not normal (2). They mentioned“31 matched (age and sex) healthy subjects from the Arak Blood Transfusion Center were enrolled.” If they were paired match, the analysis should be based on the same method (paired) (2). If not, it is necessary to explain. Moreover, selecting such controls from IBTO induces healthy worker bias. As, we know only healthy people are permitted to donate blood in Iran. Such people are selected after a closed questionnaire-based interview. Hence, healthier people will be selected. Therefore, basic chance of any value of variables like LI-2, IL-4, IL-10 and IFN-gamma is lower than the real rate in the general population. As a result, there is a higher chance of significant difference between control groups in comparison with other cases. In addition, it is not necessary to have a control group with equal number in comparison with cases. Authors can increase the number of their controls for increasing the power of the study. According to your title and objectives, it is not important to compare IFN-gamma with IL-2, 4 and 10 in HCV treated patients as they have done in Table 1. It is important to compare INF-gamma between cases that were treated for HCV, untreated for HCV and healthy controls. “There was no relationship found between serum cytokines levels, HCV genotypes and possible route of HCV acquisition”. These findings are not reliable due to low sample in each subcategory of HCV genotypes. The power of the study should have been reported for each of these comparisons. We should avoid doing different comparisons which are not among our main goals specifically when the sample size in subcategories is not adequate. This adequacy can be determined using power for each comparison. Moreover, we should have suitable control groups for these accessory comparisons to avoid biased results. Even high power is not sufficient without considering methodologically sufficient control groups for such comparisons.
